# Contributions of Civil Society Organizations to the Provision of Sexual and Reproductive Health Services to Vulnerable Populations in Latin America

**DOI:** 10.1007/s10900-025-01499-x

**Published:** 2025-07-18

**Authors:** G. Nigenda, S. J. Cedeño-Tapia, Z. Aranda, M. Santiváñez, P. Aristizabal, A. Matallana

**Affiliations:** 1 Faculty of Nursing and Obstetrics, National Autonomous University of Mexico, Mexico City, Mexico; 2 Universidad de El Salvador, San Salvador, El Salvador; 3 Independent Consultant, Mexico City, Mexico; 4 Management Sciences for Health, Lima, Peru; 5 Iztacala Faculty of Higher Studies, National Autonomous University of Mexico, Hab. Los Reyes Ixtacala, Barrio de los Arboles/Barrio de los Héroes, Tlalnepantla de Baz, 54090 Mexico City, State of Mexico Mexico; 6 Public Health Institute, Universidad Javeriana, Bogotá, Colombia

**Keywords:** Latin America, Health care delivery, Reproductive rights, Health equity, Civil organizations, Public health

## Abstract

The involvement of civil society organizations in sexual and reproductive health (SRH) programs in Latin America began to expand during the health sector reforms era. These organizations have undergone significant changes over the decades, being able to collaborate with government agencies, the private sector and international agencies with the commitment to address the specific needs of vulnerable populations in the countries. We provide a synthesized and analytical description of the experiences of 17 civil organizations that carry out actions in the field of SRH in five Latin American countries. The aim is to understand the variety of organizational models, the populations served, the funding, and the activities they perform in the SRH field. A multi-case study focused on 17 organizations that primarily carry actions on SRH, especially for vulnerable populations in Latin America: Colombia, Guatemala, Ecuador, Mexico, and Peru. Data collection was carried out in two stages: gathering preliminary information and semi-structured interviews with 55 members of the organizations. The analysis was based on categories constructed prior to the process. These are described through four categories: Characteristics of target populations; Relationship with government structure and other interested actors; Activities Carried Out in Response to SRH; Achievements and scope of civil organizations in sexual and reproductive health and reproductive rights. Civil society organizations in the studied countries are essential within health systems, acting as agents of equity, expert service providers, and with a strong commitment to their target populations. In this study, they encountered several financial and political challenges. Their ability to adapt and the recognition they receive from the populations they serve stands out. Civil organizations not only fill significant gaps in the provision of SRH services but also become catalytic forces for social change and equity. It is essential to develop funding mechanisms and support policies that allow their continuous operation, expansion, and informed participation in decision-making processes in Latin America.

## Background

In the last 30 years, the countries of Latin America and the Caribbean have undergone important changes in their quest to guarantee appropriate sexual and reproductive health services for their populations. The defining moment that generated a new dynamic of care was the signing of the Millennium Development Goals (MDGs), since all the countries that signed this initiative committed themselves to sexual and reproductive health care goals. Particularly in its goal 5, the MDGs included the reduction of maternal mortality by 2015 to 75% of the value obtained in 1990 [1].

The reduction of maternal mortality implied designing and implementing specific programs to achieve this goal, which included an increase in funding with innovations in operationalization and an increase in the availability of trained personnel for direct care of the population. The programs included not only childbirth care, but also the reinforcement of sexual and reproductive health (SRH) promotion and education tasks [2]. In some cases, the programs contained strategies for the management of social determinants through intersectoral agreements [3].

Among the cases to be highlighted are “Arranque parejo en la vida” program created in Mexico in 2001 [4], the “Programa de Humanización de la atención Pre-natal y el Nacimiento”, created in 2000 and which later gave rise to the Red Cigüeña in Brazil [5], the “Modelo de vigilancia de la morbilidad materna extrema” which started in Colombia in a pilot phase between 2007 and 2008 [6], and which in subsequent years expanded geographically and institutionally. Peru strengthened in 1994 the Maternal and Perinatal Health Program and in 1999 the Family Planning Program which contributed to the development of the “National Strategic Plan for the Reduction of Maternal and Perinatal Mortality 2009–2015” in order to optimize the use of resources and consolidate actions towards the reduction of maternal and perinatal mortality. Thus, several Latin American countries joined these regulatory and strategic changes in order to strengthen the sexual and reproductive health of their populations, in accordance with international strategies. However, the structural determinants of health existing in most countries in the region, including social inequality and ethnic and class discrimination, reduced the impact of these programs [7]. In fact, no country in the region achieved the maternal mortality target established in the MDGs [8]. Countries such as Costa Rica, Uruguay, Chile and Cuba are not included in this group, mainly because the maternal mortality ratio was at similar levels compared to regions and countries with higher income levels before the MDGs were declared [8].

It is important to consider the existence of population groups with specific needs that did not receive the services expected to meet their needs. Among these groups are indigenous populations [9], African Americans [10], adolescents [11], migrants [12] and LGTBI groups[13]. In each case, the challenges to reach these populations were diverse, including socioeconomic, cultural and geographic barriers, as well as those related to the public supply of health services such as low budgets, institutional bureaucracy and low quality of care, among others.

These programs were implemented in the context of the health sector reforms that expanded in most of the countries of the region in the last decade of the twentieth century, which sought to extend coverage, improve efficiency in the use of financial resources and increase the quality of care [14]. The reforms proposed, as a starting point, that public institutions were characterized by obsolete management mechanisms and low levels of resources, making it necessary, among other things, to innovate with new management models from the private sector and even to incorporate the participation of this sector in the financing and supply of services. It is at this juncture that civil society organizations have expanded their participation in some countries, and in others they had a pioneering role [15].

These organizations, also known as the third sector, have played an important role both as independent service providers and as partners of public institutions, not without opening debates in the countries on the importance of their participation [16]. Their origins, motivations, management and financing models, as well as their achievements, are very diverse. In the field of sexual and reproductive health (SRH) they have found a privileged space for participation and, at certain times, have managed to expand their influence not only geographically, but also in relation to their activities. In other cases, they are motivated by a vision of reproductive rights and in others by the defense of more conservative positions.

This article presents an account of the participation of a set of civil society organizations in five Latin American countries and analyzes the difficulties of their operation and achievements in the provision of SRH services. In particular, it compares the different management models, operational approaches, funding sources and strategies for their sustainability, as well as the expansion of their activities. The report describes the stages through which the organizations have passed in their relationship with the government and the adjustments they have had to implement to ensure their continuity.

## Methods

A multiple case study approach was adopted [17]. This emphasizes description, explanation, and critique, using clear boundaries and flexibility to define the cases [18, 19]. The study encompassed 17 non-profit civil organizations, operating across five Latin American countries: Colombia, Guatemala, Ecuador, Mexico, and Peru. These organizations primarily focus on delivering sexual and reproductive health services to vulnerable populations [20]. The services they provide span direct care, education/counseling, financing, research, and policy advocacy.

In the selection of the organizations, the maximum variety of experiences was represented, combining the criteria of work on SRH issues in vulnerable populations; those with decades of participation and others with recent participation in the field; with broad social recognition and with restricted recognition; with national or state/departmental/provincial coverage and with local coverage; those who reported having a social business model and others with a non-profit social model. Three organizations were chosen per country, except in Guatemala where the variety of organizations allowed the election of five.

The information collection was carried out in two stages. In the first stage, preliminary information was collected through the organizations'websites and representatives were contacted to carry out informal consultations via Zoom or in person. This first stage allowed us to select the organizations that would be included as cases. In the second stage, instruments to collect information were designed and applied to managers and operational staff of each organization. Instruments led to inquire about the services offered, scope, type of population served, organizational model, among others. When possible, group interviews were carried out with the informants to generate an interaction that allowed contrasting points of view among the participants. In other cases, the interviews were individual. A total of fifty-five informants (between two and five per organization) were interviewed. All of them gave informed consent prior to the interview.

All interviews were audio-recorded and transcribed verbatim prior to analysis. Each interview was assigned an identification code in order to maintain the anonymity of the participants. Analytical categories were constructed to guide the organization and interpretation of the data. The primary categories were: (a) history of the institution, (b) services offered, (c) organization, (d) financing, (e) achievements, and (f) other activities (in addition to the provision of services). Each researcher analyzed the information derived from their country and wrote a report containing a description of the findings. Once the individual country reports were generated, the research team met in Lima, Peru, in November 2022, to conduct a comparative and interpretive analysis of all the cases included in the study. The integrated findings of the 17 cases are presented in this study.

The relevant information from the organizations participating in the study is integrated below (Table 1).

**Table 1:** Characteristics of the organizations included in the study

Country	C	C	C	E	E	E	G	G	G	G	G	M	M	M	P	P	P
Organization	1	2	3	1	2	3	1	2	3	4	5	1	2	3	1	2	3
Public financing	✓	x	x	x	✓	✓	x	x	x	✓	x	✓	x	x	x	x	x
Private financing	✓	✓	✓	✓	✓	✓	✓	✓	✓	✓	✓	✓	✓	✓	✓	✓	✓
National coverage	✓	✓	x	✓	✓	✓	✓	✓	x	✓	x	x	x	✓	✓	✓	✓
International connections	✓	x	x	✓	✓	✓	✓	✓	✓	✓	✓	✓	x	✓	✓	✓	✓
Focus on the poor and vulnerable groups	✓	✓	✓	✓	✓	✓	✓	✓	✓	✓	x	✓	✓	✓	✓	✓	✓
Focus on indigenous communities	x	x	✓	x	✓	✓	✓	x	✓	x	x	✓	✓	✓	x	x	x
Focus on marginal urban or rural areas	✓	✓	✓	✓	✓	✓	✓	✓	✓	x	✓	✓	✓	✓	✓	✓	✓

**Codes:** “✓” indicates"Yes";"✗"indicates"No". C = Colombia, E = Ecuador, G = Guatemala, M = Mexico, P = Peru. 1, 2, etc. represent different organizations within the respective countries

## Results

The findings are described through four categories: (1) characteristics of target populations, (2) relationship with the government structure and other interested actors, (3) activities carried out in sexual and reproductive health care, (4) achievements and scope of civil organizations in sexual and reproductive health and rights. The categories are described below (Table 2).

**Table 2:** Participating organizations by country and main characteristics

Country	Name of the institution	Type	Main characteristics
COLOMBIA	(C1) Profamilia	Non-for-profit. Civil society	Profamilia is an organization focused on services provision of sexual and reproductive health and research. It was founded in 1965. It also promotes sexual and reproductive rights in 45 communities across the Colombian territory
	(C2) Red SOMOS	Non-for-profit. Civil society	Organization dedicated to the promotion of sexual and gender diversity and sexual and reproductive rights. Founded in 2007 aims at empowering communities through projects in more than 39 Colombian cities and municipalities
	(C3) Fundación Baylor	Non-for-profit. Industry-related	This organization renders sexual and reproductive health services to population living in the Guajira Department desertic area since 2015. It receives financial support from social responsibility type of for-profit enterprises related to the oil industry
GUATEMALA	(G1) Fundación Carlos Slim (CARSO)	Non-for-profit. Private foundation	This is a key actor of the development of the public–private Mesoamerican Health Initiative where all Central American countries and the State of Chiapas, Mexico participate. The Initiative started operations in 2011 and is one of the multi—organization (including Gates Foundation, IDB and governments) with highest impact in the region, specially targeting mother–child health. Fundación CARSO was founded in Mexico in 1986
	(G2) APROFAM	Non-for-profit. Civil society	This organization is the most important provider of sexual and reproductive health services in Guatemala. Founded in 1964, combines social development projects with the provision of low-cost sexual and reproductive health services for the whole population
	(G3) Funcafé	Non-for-profit. Industry related	Founded in 1994, this organization represents the institutionalized social branch of the coffee industry in the country, representing 37% of agriculture sector and contributing to 25% of Guatemala GDP. Their services focus on health, education and food security reaching most of the national territory
	(G4) Alas Guatemala	Non-for-profit. Civil society	Alas was founded in 2001 and is the second largest provider of modern contraceptive methods in the country. Its target population is that with highest levels of marginality and vulnerability. It is characterized by its holistic approach to sexual and reproductive health issues through the combination of services provision and education, promoting the active participation of communities
	(G5) Centro para el Desarrollo Humano (CDH)	Non-for-profit. Industry-related	CDH was born in 2011 from the agreement between the Guatemalan agroindustry (AgroAmerica) and the University of Colorado. Its approach to the sexual and reproductive health field is mainly through an integral educational program in sexuality for adolescents, the provision of ante-natal care, delivery and puerperium and well as the distribution of modern contraceptive methods
ECUADOR	(E1) APROFE	Non-for-profit. Civil society	It was constituted in 1965 and emphasizes the importance of family planning, the prevention of sexually-transmitted diseases and the guarantee of the highest possible level of quality of care and a gender perspective. Its objective is to provide specialized services by means of the use of highest technological developments available in the country. It works with several vulnerable communities. It offers services to diverse populations based on a public–private agreement
	(E2) Fundación PrevenSud	Non-for-profit. Civil society	Founded in 2009, its general objective is to develop a preventive model through the incorporation of information, educational and communication perspectives in order to generate participation, learning and the application of sexual and reproductive rights. It works with community leaders both governmental and from indigenous communities. It also works with school teachers and vulnerable groups
	(E3) Fundación Sendas	Non-for-profit. Civil society	Founded in 1993, its social purpose is to promote alternatives of development at local, regional and international levels. Its objective is to promote incidence in public policy to guarantee sexual and reproductive health rights. It is specialized in gender, development and environment. Sendas has national coverage and works in agreement with public agencies, to provide services and to carry out research
MÉXICO	(M1) CIMIGEN	Non-for-profit. Civil society	This is a center for perinatal care, it carries out research and training of human resources in areas of maternal care and primary health care. It was founded in 1978 and it has a national-regional scope
	(M2) Partners In Health	Non-for-profit. International civil society	PIH Mexico is located in the Sierra Madre highlands in the State of Chiapas since 2011. It is affiliated to PIH Boston. It works in collaboration with the State and National Ministries of Health. It administers 10 first-level of care health centers and one birthing center in Angel Albino Corzo municipality. It provides sexual and reproductive health services, among other types of services, as well as research and training
	(M3) MEXFAM	Non-for-profit. Civil society	Since 1965 MEXFAM has contributed to improve the quality of life of vulnerable populations through sexual and reproductive health programs and services. It has national scope by means of 9 centers deployed across the country
PERÚ	(P1) PROMSEX	Non-for-profit. Civil society	Legally registered in 2005. Its mission is to support women and adolescents about their sexuality and autonomy of reproductive decisions, dignity and justice. It reaches vulnerable groups with particular scope on higher poverty regions
	(P2) Movimiento Manuela Ramos	Non-for-profit. Civil society	Legally registered in 1978 its purpose is to contribute to the effective observance of women’s rights and to eliminate any type of discrimination and violence, from an integral democratic development perspective. It has national scope with special presence in regions with vulnerable populations
	(P3) Liga contra el cáncer	Non-for-profit. Civil society	Since 1950 its work has been based on a primary and secondary care approach, defining lines of action to sensitize on the relevance of early detection of cervical, breast and other female cancers. It also aims at facilitating the access of low and medium income women to screening tests by providing low-cost services

Source: Author’s condensation of data

### Characteristics of Target Populations

All organizations are committed to the health of vulnerable populations and base their participation on a set of values that generates moral commitment to the populations they care for, permanently being subjected to great economic and material needs, lack of information about their SRH rights and reduced access to SRH services. These populations generally benefit from this offer as they found the offer of the ministries of health with significant drawbacks to its access and effective utilization.

Thus, specific populations have been targeted by organizations, all of them considered to be vulnerable in different dimensions. Most organizations serve multiple vulnerable groups. For example, Red Somos in Colombia, PROMSEX in Perú, APROFE and SENDAS in Ecuador primarily focus on LGTBI individuals, migrants, and survivors of gender-based violence.

One key vulnerable group is that of adolescents. In all countries at least one organization was identified to be committed to work with this group. Although no organization is specialized only in these populations PROFAMILIA in Colombia, APROFE and PREVENSUD in Ecuador, APROFAM and Funcafe in Guatemala, MEXFAM in Mexico, PROMSEX and Movimiento Manuela Ramos in Perú, display specific programs. The commitment of Alas in Guatemala to the adolescent population is not limited to the provision of services specifically oriented to them, but also includes organizing and training groups of adolescents to provide information to peers in their own communities. For most of these organizations, adolescents were not a target population in their beginnings, but it has been incorporated over the recent years as it has been recognized as a complex social priority.

LGTBI as a vulnerable population is the target of several organizations although with different approaches depending on the country. As in the case of adolescents, several organizations work with LGTBI groups but only a few consider themselves to focus on them specifically. In Perú PROMSEX and Manuela Ramos, Red Somos in Colombia target these populations, consider that the SRH of these groups has been made invisible or forgotten due to social stereotypes and subject to mistreatment by many other social actors, including the government.

Organizations that particularly work with indigenous populations such as Fundación Baylor in Colombia, Prevensud in Ecuador and Partners in Health in Mexico generally adapt to the characteristic features of ethnic groups to provide culturally-appropriate services that consider aspects regarding their ideologies and practices in topics such as reproduction, pregnancy and childbirth.

### Relationship with the Government Structure and Other Interested Parties

The set of selected organizational models shows a wide diversity. All institutions included in the study have an NGO status. Pioneering institutions were founded in the 1960 s and 1970 s and are still in operation including PROFAMILIA in Colombia, MEXFAM in Mexico, APROFAM in Guatemala, APROFE in Ecuador, Movimiento Manuela Ramos and Liga Contra el Cáncer in Perú. They have been able to accommodate health systems models and changes over time, particularly to the reforms that occurred in the 1990s. Most of these pioneer institutions were able to partner with central and local governments, private insurers and international donors. Two cases should be highlighted. The first is PROFAMILIA that was able to register as a Services Provider Institution (IPS in Spanish) after the 1990 s health reform and contract with system’s financing agencies to provide services to their target populations. The second case is APROFE in Ecuador that contracts with several private insurers as well as with the government to provide a broad menu of services to populations covered by the insurers.

Organizations established after the 1990 s faced a different financial environment as they were born in a more favorable context characterized by a greater openness of the government towards this type of organizations and the capacity of the organization to respond to the needs of underserved populations. However, in the last decade, they have been facing financial difficulties that have led some of these organizations to consider their future existence. Nonetheless, pioneer and more recent organizations have demonstrated resilience by moderately or radically modifying their organizational precepts to achieve the ultimate goal of adapting to changes in the environment and maintaining the provision of care to underserved populations in SRH.

A type of organizations that is relevant to highlight are those that have linkages with the agroindustry as in Guatemala. Although organizations focus on the provision of vulnerable populations, they limit their coverage to the populations living in those regions, mainly rural, where the industrial production of agricultural products happens. This is the case of Funcafe, and Centro para el Desarrollo Humano. Worth is to say that these institutions consider sexual and reproductive health activities as one more item of their menu of activities as they also include educational and nutritional programs, among others.

Also, an increase in international participation was observed through United Nations agencies, development banks and private foundations that represent sources of resources available to civil organizations in the field of SRH. However, as national economies have grown, many of these foreign foundations have closed offices in various Latin American countries to seek to invest their resources in low-income countries. This has forced civil organizations in the countries studied to develop new financial strategies, such as charging for services according to the population's ability to pay and the implementation of private subsidies to continue covering the most vulnerable groups.

### Activities Carried Out in the Care of Sexual and Reproductive Health

Most of the organizations studied were created to carry out a specific type of activity, but over time these activities have expanded for reasons of subsistence and/or representativeness in the institutional ecosystem. In this sense, it is possible to classify organizations by their original main activity into:

- *Organizations providing direct services:* mainly SRH services, such as perinatal and maternal care, family planning, etc.
- *Organizations dedicated to health education and/or research:* oriented towards education and research in health areas for demographic groups, such as young people or vulnerable populations.
- *Organizations dedicated to the incidence and promotion of citizen exercise in SRH and rights:* focused on promoting SRH rights in groups most vulnerable to access to SRH care and demanding their prioritization on the political agenda.
- *Organizations Providing Logistical Support and Resources for SRH Care:* These organizations provide human, financial, and technical resources, infrastructure, and other means to strengthen sexual and reproductive health (SRH) initiatives. Their contribution expands service coverage, enhances the operational capacity of healthcare centers, and ensures the sustainability of programs.

It is worth noting that the institutional ecosystem in Guatemala differs from that of other countries due to its high maternal and infant mortality rates. In this country, several international foundations have begun to withdraw, creating financial challenges for local organizations. However, there are still important efforts to articulate a joint program between all agencies and in this effort the Inter-American Development Bank, the Carlos Slim Foundation, the Bill & Melinda Gates Foundation, and the AbbVie Foundation, among others, have played a relevant role. Moreover, due to the economic significance of export agriculture, large companies engaged in coffee, bananas, sugar and palm production have established civil organizations responsible for the health care of their workers and families, with a special focus on SRH.

The diversification of activities is a relatively recent phenomenon. Organizations with a long history, such as Profamilia in Colombia, Aprofam in Guatemala, Mexfam in Mexico, APROFE in Ecuador or the Manuela Ramos Movement in Peru, have remained relatively faithful to their initial approach. For more than four decades, these organizations have provided services linked to family planning and/or reproductive health care. Meanwhile, more recently founded organizations have focused on citizen education, research, and policy advocacy, with the latter becoming a prominent feature of newly established organizations. For example, SENDAS and PREVENSUD in Ecuador have promoted projects aimed at improving SRH through strategic partnerships that amplify their impact. They have also worked in coordination with ministries, Decentralized Autonomous Governments (DAGs), and Rights Protection Councils to ensure the integral development of children and adolescents, as well as to prevent xenophobia and human trafficking in contexts of human mobility. The origin of these new lines of intervention arises from the international meeting in Cairo, where a vision of rights in this field was proposed for the first time.

### Achievements, Failures, and Scope of Civil Organizations in Sexual and Reproductive Health and Rights

Based on the diversity of the organizational models of the cases studied, recurrences were found in various achievements obtained during the operation time. It is possible to identify various achievements of civil organizations in the field of SRH in the countries studied. These include:

- *Coverage of vulnerable populations:* Although limited, its presence is frequently the only SRH care option for vulnerable groups particularly those cases that work with geographically isolated and rural populations (e.g., Fundación Baylor, Partners in Health and PREVENSUD)
- *Transfer of the rights and gender agenda:* Its capacity to expand this agenda is more effective than that of government institutions, mobilizing various sectors of society to place problems pending resolution on the public agenda (e.g., PROMSEX, SENDAS and Movimiento Manuela Ramos).
- *Channeling public and private resources:* Organizations are capable of optimizing the resources they channel into SRH interventions that respond to the needs of the communities they serve (e.g., PROFAMILIA, CIMIGEN, Red Somos).
- *Intermediation between the government structure and the population:* They act as a bidirectional bridge between citizens and government institutions, especially in contexts of vulnerability through focused attention to vulnerable groups, technical collaboration mechanisms and cooperation in preferred interventions. Preventive promotional SRH and rights in specific areas (e.g., Movimiento Manuela Ramos, PROFAMILIA, APROFE).
- *Contribution to generate evidence:* Share with government agencies and citizen networks to make more efficient decisions in the management of SRH care (e.g., Liga contra el Cáncer, Partners in Health).

It should be considered that in these five aspects, the achievements are limited but do not lack relevance. Although the coverage of vulnerable populations is restricted, since the majority of the organizations studied have areas of focused intervention, the fact that they remain in communities with the greatest vulnerabilities represents a fundamental support for populations that cannot be obtained in a timely manner from government institutions, which is why they offer essential support that complements or replaces State action.

Without a doubt, the expansion capacity of the SRH rights agenda by civil organizations is much greater than that of government institutions since the mentioned transfer is not only carried out in the direct provision of services, but also in community spaces, academic institutions and in government bodies such as the ministries and legislative chambers of the countries.

The channeling of resources represents a great advance, since civil organizations, through intermediation, are generally capable of transforming resources from public or private sources into the provision of educational and informational services and interventions for the communities they serve. Civil organizations not only channel financial resources, but also know the specific demands and needs of the populations they serve. Particularly they perform in contexts of social vulnerability and are capable of bringing over populations’ demands to government policy decision-making spaces, allowing them to formulate more effective strategies to address issues such as poverty, marginalization and inequality. This intermediation is therefore bidirectional.

## Discussion

Civil society organizations represent an essential pillar within the health systems of each of the study countries, since they actively support the coverage and provision of SRH and family planning services [21], especially in marginalized areas. Government interaction with these organizations is not steady nor trustworthy in most of the studied countries while the access of vulnerable populations to public health services, including SRH, is seriously restricted. Furthermore, these organizations are not naturally inserted into the organizational network, but rather, their participation must be incorporated through mechanisms that include contracting and inter-institutional agreements [22]. Furthermore, their work through advocacy actions tends to put priority needs in government decisions in the political agenda.

The sample of organizations included in the study shows that some of them have been present for decades in the provision of SRH services. The oldest ones focused their attention on specific activities, mainly the provision of services, and the most recent ones have managed to diversify these activities including training, research and advocacy. It is noteworthy that political advocacy activity is currently carried out, under different modalities, by practically all organizations, both directly and indirectly. This interest is motivated by the influence that the Cairo Agenda generated on the discourse in the field of SRH [23]. The inclusion of a language of rights has permeated agencies to identify problems inherent to its violation in the provision of services by the state [24].

It is also possible to identify two main types of organizations in relation to their management autonomy: (a) those that establish full autonomy from their financiers and (b) those that were created by the financiers and that adjust to their guidelines. In general, the first come from social movements that at some point considered participating by providing direct services to the populations. The organizations that were generated by the intervention of their funder generally adhere to the guidelines for which they were generated and cover the populations of interest of the funder [25]. Within this group, there are organizations in Guatemala and Ecuador that cover populations that participate in the national agroindustry.

Organizations in general have experienced the effect of the withdrawal of philanthropic foundations from their countries, which has reduced their financial availability [26]. This has led them to seek new financial mechanisms, one of them being direct charging for services provided, which generally goes against their tradition of not charging the populations they serve. The objective is to survive as an organization and return funding in services of great value to their target populations. Organizations with direct financial dependence, although they have faced this new financial environment, maintain stability in their finances [27].

The organizations studied act as agents of equity in the system [28]. All the organizations studied had the objective of providing care, information, and counseling services to vulnerable populations, including indigenous people, adolescents, and LGTB, mainly. These populations frequently receive little protection from the State for different reasons. Their care can be very expensive given their geographical location, they can potentially reject the State's offer, they do not represent a real priority for government institutions, among others [29]. Civil organizations reach these spaces to provide care by mobilizing financial, pharmacological, human and technological resources.

Financial stringency has not been the only adverse factor that agencies have faced in recent years. Some of them have managed to maintain financial and political support from the government for many years, given their prestige and national and international recognition. Others, however, have resented the restriction of public budgets due to austerity policies or because they represent uncomfortable partners by questioning the logic with which these policies are designed and implemented [30]. Thus, the financial and political environments that face the majority of organizations are unstable, so they need to permanently incorporate management innovations including the search for financial options. In most cases, they are able to subsist for long periods, but the risk of collapse is permanent. In May 2024, CIMIGEN an emblematic organization with decades of presence in Mexico, was shut down as their main donors were not able to continue subsidizing the program [31].

The civil organizations that participate in different activities in the production of SRH services in the Latin American region have built their history based on the resilience they have shown for decades [32]. Further, they have to build their future on a daily basis as mid- and long-term planning is uncertain. Their systemic role is unquestionable since they fill gaps that public institutions leave, promoting the rights-based approach at different levels of the public institutional structure and among populations. However, the next few years are crucial for their survival as the political and financial environment has changed drastically after the last US election. Initially, the demographic trend marks the continuity of the reduction in fertility and the increase in aging [33], followed by financial and political environments that may not be favorable in the short and medium term [34]. However, the recognition by the populations they serve and the benefits they obtain, as well as the adaptive capacity of these institutions, raises the need for their presence beyond future demographic, political and financial changes. It will be important to observe its role as disseminators of SRH rights and from its advocacy actions as a new role that allows exploring the roots of the problems that will lead us to recognize its role as an agent of governance in SRH.

### Study Limitations

One of the main limitations of the study is selection bias. Participants were recruited by the 17 participating NGOs, which selected individuals according to their own criteria, potentially incurring selection bias. As some of the interviews and the FGDs were conducted online, this could have left out of the study informants from the selected organizations who were based in areas without access to information and communication technologies. The fact that participants were active employees of the NGOs they were talking about could have limited the information shared by the participants, especially that which could compromise the image of the institution or its relationship with public institutions.

## Conclusions

Civil society or non-governmental organizations in the countries studied, not only fill important gaps in the provision of SRH services, but also become catalytic forces for social change and equity. However, they face financial challenges and political positioning that threaten their long-term sustainability. As these countries move toward demographically different futures, the role of these organizations becomes even more crucial, requiring funding mechanisms and support policies that allow for their continued operation, expansion, and informed participation in decision-making processes in resolving issues gaps in SRH in Latin America where great inequities remain. This research highlights the urgent need for sustained commitment and multi-sector collaborations to ensure that these organizations continue to be key actors in promoting equitable and effective SRH and rights advocacy in the region.

## Data Availability

The datasets used and/or analyzed during the current study are available from the corresponding author on reasonable request.
